# Efficacy and safety of apremilast and phototherapy versus phototherapy only in psoriasis vulgaris

**DOI:** 10.1111/1346-8138.16566

**Published:** 2022-09-24

**Authors:** Akimichi Morita, Yukie Yamaguchi, Chiharu Tateishi, Kyoko Ikumi, Aya Yamamoto, Haruna Nishihara, Daisuke Hayashi, Yukihiko Watanabe, Yuko Watanabe, Ayano Maruyama, Koji Masuda, Daisuke Tsuruta, Norito Katoh

**Affiliations:** ^1^ Department of Geriatric and Environmental Dermatology Nagoya City University Graduate School of Medical Sciences Nagoya Japan; ^2^ Department of Environmental Immuno‐Dermatology Yokohama City University Graduate School of Medicine Yokohama Japan; ^3^ Department of Dermatology Osaka Metropolitan University, Graduate School of Medicine Osaka Japan; ^4^ Department of Dermatology Kyoto Prefectural University of Medicine Graduate School of Medical Science Kyoto Japan

**Keywords:** apremilast, phosphodiesterase‐4 inhibitor, phototherapy, psoriasis, Psoriasis Area and Severity Index

## Abstract

Phototherapy and apremilast (oral phosphodiesterase‐4 inhibitor) are well‐known in the treatment of moderate to severe psoriasis vulgaris. However, current evidence on the efficacy and safety of their combination is not sufficient. This multicenter, randomized controlled study compared the efficacy and safety between phototherapy as monotherapy and phototherapy and apremilast as combination therapy in patients with psoriasis vulgaris. Patients with moderate to severe psoriasis vulgaris were assigned to combination (*n* = 29) and monotherapy (*n* = 13) groups. All patients underwent an 8‐week phototherapy regimen comprising irradiation with narrowband UV‐B. The patients in the combination group were also administered 10 mg to 60 mg of oral apremilast. We evaluated the improvement percentage based on the Psoriasis Area and Severity Index (PASI) score from baseline to week 8. Additionally, we evaluated the percentage of patients who achieved ≥75% improvement; changes in body surface area (BSA) and scores of EuroQol 5‐dimensions 5‐level, Dermatology Life Quality Index, and visual analog scale for pruritis from baseline to 4 and 8 weeks; and adverse events. Compared with the monotherapy group, the combination group had significantly lower PASI scores at 4 and 8 weeks and more patients who achieved a PASI score improvement of ≥75% at 8 weeks. Both groups exhibited a significant decrease in BSA; at 8 weeks, no significant difference was observed between the two groups, although the combination group tended toward a greater reduction in BSA. The intergroup differences in the changes at the three time points were not significant. Adverse events were more frequent in the combination group than in the monotherapy group. Our findings suggest that an 8‐week combined apremilast and phototherapy regimen may not be adequate in patients for improvements in their subjective assessment of psoriasis, and longer treatment periods may be necessary.

## 1 INTRODUCTION

Inflammatory cytokines, mainly tumor necrosis factor α (TNF‐α); interleukin (IL) 1, IL‐6, IL‐8, and IL‐36; and T helper 17‐related cytokines (IL‐17, IL‐22, and IL‐23) are involved in the pathogenesis of psoriasis.^1^ The production of inflammatory cytokines is suggested to involve intracellular signaling through the nuclear factor kappa B transcription factor.^2^ Patients with mild to moderate psoriasis are topically treated with corticosteroids and active vitamin D3 derivatives. Systemic treatment is usually considered for patients with moderate to severe psoriasis, and it includes oral medications^3^, ^4^ such as apremilast, cyclosporine, and etretinate and phototherapies, such as narrowband UV‐B (NB‐UVB), excimer light, and psoralen UV‐A.^5^ Biologics may be used in conjunction with these treatments in severe cases or refractory sites.

Apremilast, a small‐molecule oral phosphodiesterase‐4 (PDE4) inhibitor that regulates a network of inflammatory and anti‐inflammatory mediators in cells, was first approved overseas in 2014 and in December 2016 in Japan. Apremilast is effective in moderate to severe plaque psoriasis (psoriasis vulgaris) that has not responded adequately to topical and other treatments.^6^ By inhibiting PDE4, apremilast suppresses the inflammatory response by increasing intracellular cyclic adenosine monophosphate levels and regulating the expression of inflammatory cytokines and chemokines, such as TNF‐α, IL‐23, CXCL9, and CXCL10.^7^, ^8^, ^9^

The combination of cyclosporine and phototherapy is not usually recommended because of the immune suppressive capacity of cyclosporine. Although the combination of biologics and phototherapy is not currently recommended for psoriasis, targeted phototherapy with biologics may be indicated for psoriatic lesions that persist after treatment^5^; adalimumab (a biologic preparation) with phototherapy has been reported to be highly effective.^10^ On the basis of the different action mechanisms of apremilast and phototherapy, their combined use is expected to exhibit increased efficacy.

We compared the efficacy and safety of the combination therapy–apremilast and phototherapy–with those of the monotherapy–phototherapy–in patients with psoriasis vulgaris.

## 2 METHODS

### 2.1 Study design and treatment

This was a multicenter, randomized, open‐label, parallel‐group, active‐controlled study conducted at four institutions between November 9, 2018, and February 14, 2020. The study was conducted according to the ethical principles of the Declaration of Helsinki (2013 amended) and Clinical Trials Act (Act No. 16 of 2017) and its related notifications. The study protocol was reviewed and approved by the Nagoya City University Hospital's certified review board (number 4180001). All of the patients provided written informed consent for their participation in the study at the time of their screening visit. This study was registered in the Japan Registry of Clinical Trials system (jRCTs041180012).

With body surface area (BSA) as an allocation factor, stratified permuted block randomization was used to allocate the patients to the combination therapy group, apremilast and phototherapy, or monotherapy group, phototherapy only, in a 2:1 ratio. Apremilast was orally administered as 10 mg (in the morning), 20 mg (morning: 10 mg, evening: 10 mg), 30 mg (morning: 10 mg, evening: 20 mg), 40 mg (morning: 20 mg, evening: 20 mg), 50 mg (morning: 20 mg, evening: 30 mg) on days 1, 2, 3, 4, and 5, respectively, followed by 60 mg daily since day 6 (morning: 30 mg, evening: 30 mg). NB‐UVB (311 ± 2 nm) was administered twice a week per the phototherapy guidelines for psoriasis.^5^, ^11^ The initial dose was 50% of the minimal erythema dose or 0.3 to 0.5 J/cm^2^. The dose increment was 0.05 to 0.1 J/cm^2^ each time as required, with a maximum dose of 1.5 J/cm^2^. The treatment period was 8 weeks, and usefulness was evaluated 4 weeks after the first dose.

### 2.2 Patients

The inclusion criteria comprised the following: (1) age 20 to 80 years; (2) diagnosis of moderate to severe psoriasis vulgaris (BSA or Static Physician's Global Assessment [sPGA] score of ≥5% or ≥3, respectively); and (3) inadequate response to topical treatment. The main exclusion criteria were as follows: (1) diagnosis of psoriasis other than psoriasis vulgaris or psoriatic arthritis; (2) inadequate response to phototherapy; (3) history of cutaneous malignancy (although patients could be included if they had no recurrence in the previous 5 years); (4) high risk for carcinogenesis; (5) phototherapy with UV‐A or UV‐B radiation within 2 weeks of treatment; (6) treatment with the strongest topical steroids within 2 weeks of treatment; (7) relapse or flare‐up of psoriasis within 4 weeks of treatment; (8) cyclosporine or methotrexate treatment within 4 weeks of treatment; (9) etretinate treatment within 12 weeks of treatment; (10) biologics treatment for psoriasis within 12 weeks of treatment or treatment with secukinumab or risankizumab within 24 weeks of treatment; and (11) ineligibility as determined by the physician.

### 2.3 Study evaluations

The primary efficacy end point was the improvement rate according to the Psoriasis Area and Severity Index (PASI) score from baseline to 8 weeks of treatment. The secondary efficacy end points were as follows: (1) the PASI score and improvement rate from baseline to 4 and 8 weeks after treatment; (2) number and percentage of patients achieving ≥90%, ≥75%, and ≥50% improvements (PASI90, PASI75, and PASI50, respectively) in the PASI score; (3) change in the PASI score from baseline to each evaluation time point; (4) change in the BSA from baseline to each evaluation time point; (5) sPGA scores at baseline and at each time point; (6) percentage of patients having an sPGA score of 0 (disappeared) or 1 (almost disappeared), i.e., the sPGA 0/1 achievement rate at 4 and 8 weeks; and (7) changes in the scores of EuroQol 5‐dimensions 5‐level (EQ‐5D‐5L), Dermatology Life Quality Index (DLQI), and visual analog scale (VAS) for itchiness from baseline to each evaluation time point. For safety analysis, adverse events data were collected and evaluated.

### 2.4 Statistical analysis

The efficacy analysis was performed for the full analysis set (FAS) and per‐protocol set (PPS). For primary efficacy end point analysis, between‐group comparisons of the PASI score improvement at week 8 of treatment were performed by analysis of covariance with the allocation factor as a covariate. The primary analysis was performed using the baseline‐observation‐carried‐forward (BOCF) method to complement the missing values. As a sensitivity analysis, the analyses were performed without and with complementing the missing values using the last‐observation‐carried‐forward (LOCF) method. The secondary end points were analyzed without supplementation of the missing values.

The baseline PASI scores and percentage improvement at each assessment time were summarized for each group. Improvements in the PASI score of ≥90%, ≥75%, and ≥50% (PASI90, PASI75, and PASI50, respectively) at 4 and 8 weeks were determined and compared between the groups using chi‐square test. Within‐group and between‐group comparisons of the PASI score and BSA changes at each assessment time points were performed using paired *t* test and Student *t* test, respectively. The sPGA scores at each evaluation time point were summarized for each group, and the percentage of achieved sPGA scores was also calculated. Between‐group comparisons of the sPGA scores were performed by chi‐square test.

To assess the quality of life, the EQ‐5D‐5L scores at baseline and at each assessment time point were obtained and summarized for each group. The changes in these scores from baseline to each time point were also summarized and compared between the groups using Student *t* test. The DLQI scores were calculated at each evaluation time point, and the changes in the scores from baseline were also calculated. Group comparisons of these scores were performed using chi‐square test. The VAS scores and changes in the scores from baseline to each evaluation time were summarized; group comparisons were performed using Student *t* test. Statistical analysis was performed with SAS version 9.4 (SAS Institute Inc). All tests were performed using a two‐tailed test at a 5% significance level.

## 3 RESULTS

### 3.1 Efficacy

**FIGURE 1:**
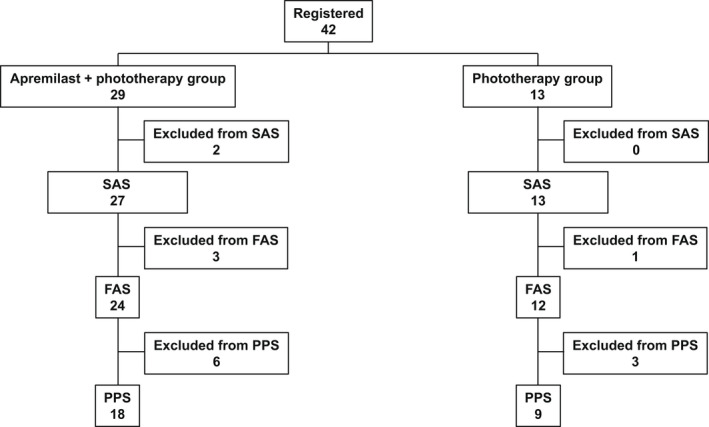
Patient disposition. FAS, full analysis set; PPS, per‐protocol set; SAS, safety analysis set.

A total of 42 patients were enrolled in the study (combination therapy group: 29, monotherapy group: 13). The safety analysis set, and number of patients included in the FAS and PPS are shown in Figure 1. The characteristics of the patients in the safety analysis set are summarized in Table 1. No statistical difference was observed between these groups.

**TABLE 1:** Characteristics of the patients

Characteristic	Apremilast + phototherapy (*n* = 27)	Phototherapy (*n* = 13)	Total (*n* = 40)
Sex			
Male	18 (66.7)	10 (76.9)	28 (70.0)
Female	9 (33.3)	3 (23.1)	12 (30.0)
Age (years)			
Mean ± SD	58.7 ± 14.3	65.7 ± 14.8	61.0 ± 14.6
Height (cm)			
Mean ± SD	164.08 ± 7.25	160.08 ± 10.51	162.78 ± 8.52
Weight (kg)			
Mean ± SD	65.35 ± 12.26	60.81 ± 16.82	63.88 ± 13.85
BSA			
<10%	11 (40.7)	5 (38.5)	16 (40.0)
≥10%, <20%	8 (29.6)	4 (30.8)	12 (30.0)
≥20%	8 (29.6)	4 (30.8)	12 (30.0)
PASI score			
Mean ± SD	12.40 ± 8.47	17.08 ± 11.15	13.92 ± 9.54
Duration of plaque psoriasis (years)			
Mean ± SD	14.56 ± 12.38	11.06 ± 10.93	13.39 ± 11.89

*Note*: Values are expressed as number (percentage) unless otherwise indicated.

Abbreviations: BSA, body surface area; PASI, Psoriasis Area and Severity Index; SD, standard deviation.

**FIGURE 2:**
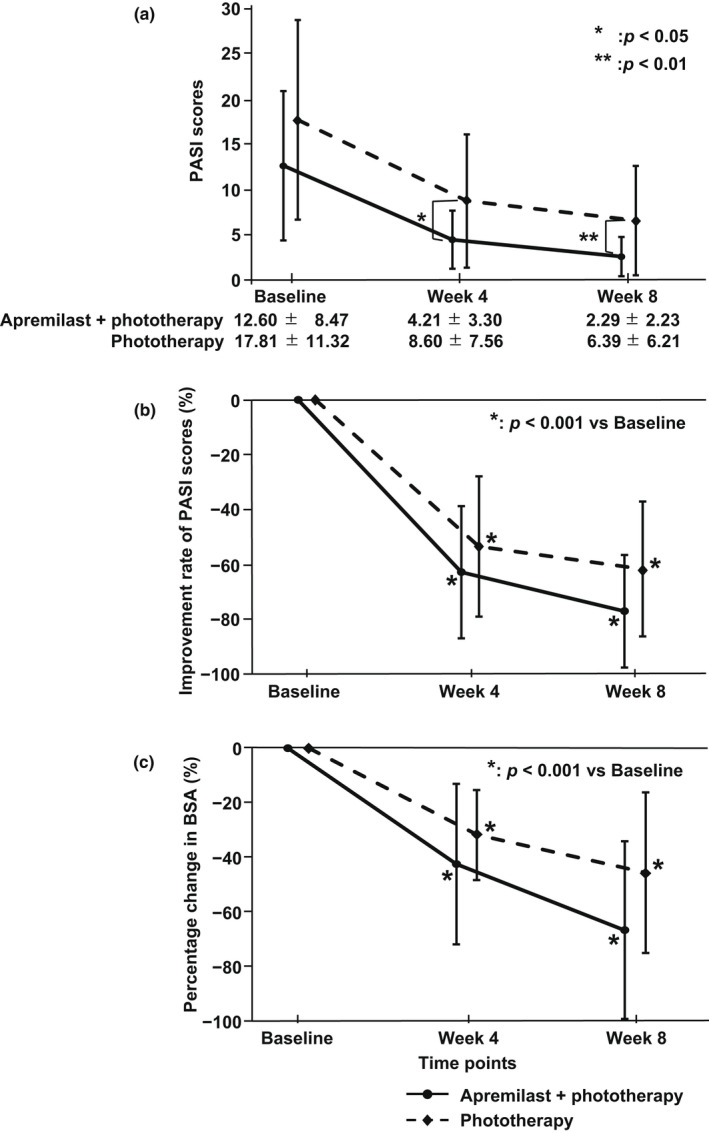
Time series variations of the Psoriasis Area and Severity Index (PASI) score and body surface area (BSA) score. (a) Changes in the PASI scores. (b) Degree of improvement in PASI scores. (c) Degree of change in the BSA scores. The round markers and solid lines represent the combination therapy group, and the diamond‐shaped markers and dotted lines represent the monotherapy group.

The PASI scores at each evaluation time point are shown in Figure 2a. The PASI scores at baseline, week 4, and week 8 were significantly lower in the combination therapy group than in the monotherapy group (4 weeks, *p* = 0.020; 8 weeks, *p* = 0.008) (Figure 2a). The improvement rates in the PASI scores are shown in Figure 2b. No intergroup differences in the PASI improvement rates were detected from baseline to each assessment time. However, within each group, the PASI score significantly decreased from baseline to each assessment time (*p* < 0.001) (Figure 2b).

**FIGURE 3:**
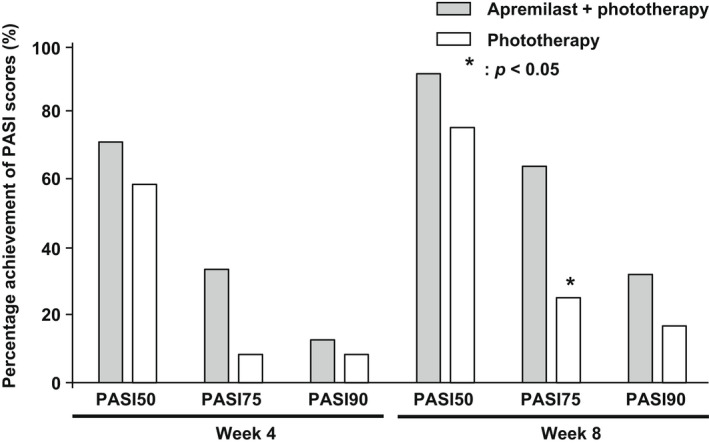
Percentage achievement of the Psoriasis Area and Severity Index (PASI) scores. The gray boxes represent the apremilast and phototherapy combination group, and the white boxes represent the phototherapy monotherapy group.

The PASI75 achievement rate tended to be higher at 4 weeks and significantly higher at 8 weeks in the combination therapy group than in the monotherapy group (*p* = 0.031) (Figure 3). No differences in the PASI50 and PASI90 achievement rates were detected between the two groups at weeks 4 and 8 (Figure 3).

The percentage change in the BSA from baseline to each evaluation time decreased over time in both groups (*p* < 0.001) (Figure 2c). The BSA change rate did not significantly differ between the two groups at 8 weeks, but the decrease in BSA in the combination therapy group tended to be greater.

**FIGURE 4:**
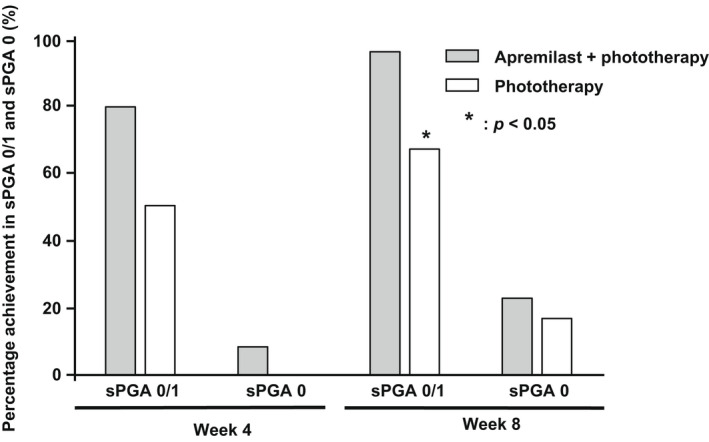
Percentage achievement in the Static Physician's Global Assessment (sPGA) 0/1 and sPGA 0. The gray boxes represent the apremilast and phototherapy combination group, and the white boxes represent the phototherapy monotherapy group.

The rate of sPGA 0/1 achievement, in which the sPGA score was 0 or 1 at each evaluation time, tended to be higher at 4 weeks and significantly higher at 8 weeks in the combination therapy group than in the monotherapy group (*p* < 0.05) (Figure 4).

A stratified analysis of the PASI improvement rate was conducted by dividing data into three strata (<10%, 10%–20%, and >20%) by the value of BSA. The PASI improvement rate in the combination therapy group did not differ between the FAS and PPS when the missing values were supplemented by the BOCF method. When the missing values were supplemented by the LOCF method or when the missing values were not supplemented, there was a trend toward a higher PASI improvement in the FAS (Table 2) and a significantly higher PASI improvement in the PPS (Table 3). In the combination therapy group, there was no effect of the percentage of BSA on PASI improvement, but in the monotherapy group, the higher the percentage of BSA, the lower the PASI improvement.

**TABLE 2:** Improvement rate of PASI score from the start of treatment to week 8 (FAS).

Complementation of missing values	Group	BSA, % (*n*)	Mean ± SD	*p* Value (intergroup)
BOCF	Apremilast + phototherapy	<10% (10)	−77.45 ± 19.36	0.374
		≥10%, <20% (8)	−52.95 ± 40.10	
		≥20% (6)	−84.01 ± 15.77	
	Phototherapy	<10% (4)	−81.03 ± 14.39	
		≥10%, <20% (4)	−53.26 ± 14.92	
		≥20% (4)	−51.47 ± 32.72	
LOCF	Apremilast + phototherapy	<10% (10)	−77.45 ± 19.37	0.060
		≥10%, <20% (8)	−72.04 ± 23.66	
		≥20% (6)	−84.01 ± 15.77	
	Phototherapy	<10% (4)	−81.03 ± 14.39	
		≥10%, <20% (4)	−53.26 ± 14.92	
		≥20% (4)	−51.47 ± 32.72	
None	Apremilast + phototherapy	<10% (10)	−77.45 ± 19.37	0.083
		≥10%, <20% (6)	−70.60 ± 27.49	
		≥20% (6)	−84.01 ± 15.77	
	Phototherapy	<10% (4)	−81.03 ± 14.39	
		≥10%, <20% (4)	−53.26 ± 14.92	
		≥20% (4)	−51.47 ± 32.72	

Abbreviations: BOCF, baseline observation carried forward; BSA, body surface area; FAS, full analysis set; LOCF, last observation carried forward; PASI, psoriasis area and severity index; SD, standard deviation.

**TABLE 3:** Improvement rate of PASI score from the start of treatment to week 8 (PPS).

Complementation of missing values	Group	BSA, % (*n*)	Mean ± SD	*p* Value (intergroup)
BOCF	Apremilast + phototherapy	<10% (9)	−80.07 ± 18.57	0.178
		≥10%, <20% (5)	−64.26 ± 38.41	
		≥20% (4)	−81.15 ± 19.40	
	Phototherapy	<10% (3)	−85.12 ± 14.50	
		≥10%, <20% (3)	−54.14 ± 18.14	
		≥20% (3)	−37.20 ± 19.59	
LOCF	Apremilast + phototherapy	<10% (9)	−80.07 ± 18.57	0.025
		≥10%, <20% (5)	−80.88 ± 13.65	
		≥20% (4)	−81.15 ± 19.40	
	Phototherapy	<10% (3)	−85.12 ± 14.50	
		≥10%, <20% (3)	−54.14 ± 18.14	
		≥20% (3)	−37.20 ± 19.59	
none	Apremilast + phototherapy	<10% (9)	−80.07 ± 18.57	0.034
		≥10%, <20% (4)	−80.33 ± 15.70	
		≥20% (4)	−81.15 ± 19.40	
	Phototherapy	<10% (3)	−85.12 ± 14.50	
		≥10%, <20% (3)	−54.14 ± 18.14	
		≥20% (3)	−37.20 ± 19.59	

Abbreviations: BOCF, baseline observation carried forward; BSA, body surface area; LOCF, last observation carried forward; PASI, psoriasis area and severity index; PPS, per‐protocol set; SD, standard deviation.

### 3.2 Quality of life

**FIGURE 5:**
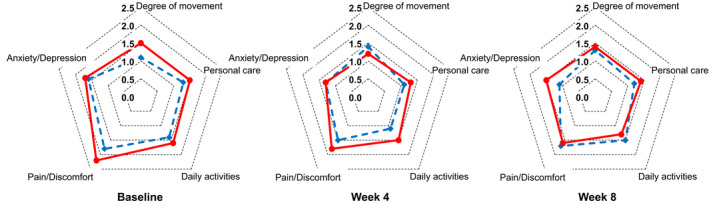
Cobweb charts of the EuroQol 5‐dimensions 5‐level (EQ‐5D‐5L) scores. The red round markers and red solid lines represent the apremilast and phototherapy combination group, and the blue diamond‐shaped markers and blue dotted lines represent the phototherapy monotherapy group.

The magnitude of changes in the EQ‐5D‐5L scores did not differ significantly between the two groups at weeks 4 and 8 (Figure 5). The DLQI and VAS scores for itchiness did not differ between the two groups in terms of a change from baseline to each evaluation time; however, these scores tended to decrease in the combination therapy group (Table 4).

**TABLE 4:** VAS score for itchiness and DLQI score at each evaluation time.

Group	Time (*n*)	VAS score	DLQI score
		Mean ± SD	Mean ± SD
Apremilast + phototherapy	Baseline (24)	27.8 ± 30.4	7.3 ± 7.4
	Week 4 (24)	13.5 ± 23.4	4.4 ± 7.1
	Week 8 (22)	12.8 ± 21.2	4.4 ± 6.8
Phototherapy	Baseline (12)	28.1 ± 29.4	6.0 ± 6.6
	Week 4 (11)	16.1 ± 18.0	3.1 ± 3.3
	Week 8 (12)	21.1 ± 23.3	3.5 ± 3.6

Abbreviations: DLQI, dermatology life quality index; SD, standard deviation; VAS, visual analogue scale.

### 3.3 Safety

Adverse events were more frequent in the combination therapy group (56 adverse events in 19 patients) than in the monotherapy group (seven adverse events in four patients) (Table 5).

**TABLE 5:** Adverse events

	Apremilast + phototherapy (*n* = 27)		Phototherapy (*n* = 13)	
	Patients, *n* (%)	Events	Patients, *n* (%)	Events
Adverse events (overall)	19 (70.4)	56	4 (30.8)	7
Gastrointestinal disorders	14 (51.9)	20	0 (0.0)	0
Soft stools	6 (22.2)	6	0 (0.0)	0
Diarrhea	4 (14.8)	4	0 (0.0)	0
Nausea	4 (14.8)	4	0 (0.0)	0
Abdominal discomfort	3 (11.1)	3	0 (0.0)	0
Infections and infestations	8 (29.6)	8	3 (23.1)	4
Nasopharyngitis	4 (14.8)	4	1 (7.7)	1
Skin and subcutaneous tissue disorders	2 (7.4)	5	1 (7.7)	1
Nervous system disorders	4 (14.8)	5	0 (0.0)	0
Headache	3 (11.1)	4	0 (0.0)	0
Musculoskeletal and connective tissue disorders	3 (11.1)	5	0 (0.0)	0
Respiratory, thoracic, and mediastinal disorders	2 (7.4)	3	0 (0.0)	0
General disorders and administration site conditions	2 (7.4)	2	1 (7.7)	1
Abnormal clinical laboratory values	2 (7.4)	2	0 (0.0)	0
Injury, poisoning, and procedural complications	2 (7.4)	2	0 (0.0)	0
Neoplasms benign, malignant and unspecified	0 (0.0)	0	1 (7.7)	1
Blood and lymphatic system disorders	1 (3.7)	1	0 (0.0)	0
Endocrine disorders	1 (3.7)	1	0 (0.0)	0
Metabolism and nutrition disorders	1 (3.7)	1	0 (0.0)	0
Cardiac disorders	1 (3.7)	1	0 (0.0)	0

The most frequently reported adverse events in the combination therapy group were soft stools (six patients, 22.2%); diarrhea, nausea, and nasopharyngitis (four patients, 14.8%); abdominal discomfort (three patients, 11.1%); and headache (three patients, 11.1%).

In addition, three serious adverse events (ileus, goiter, and complete atrioventricular block) were reported in two patients in the combination therapy group, whereas two serious adverse events (septic shock and liver cancer) were reported in one patient in the monotherapy group. A total of 29 adverse drug reactions were reported in 14 patients in the combination therapy group only; however, these were not considered as serious.

## 4 DISCUSSION

Several studies have demonstrated only moderate efficacy of apremilast monotherapy for psoriasis vulgaris. In two studies in which a 16‐week treatment with apremilast monotherapy was administered, the PASI75 achievement rate was 55.9% in 34 patients with moderate to severe disease^12^ and 41.5% in 70 patients with psoriasis vulgaris.^13^ In two Japanese studies, the PASI75 achievement rate at week 16 ranged from 23.5% to 28.2% in 254 patients with moderate to severe psoriasis vulgaris,^14^ and PASI100 was achieved in only five (11.4%) of the 44 patients with psoriasis at week 12 after treatment with apremilast.^15^ Therefore, based on the results of these clinical studies, apremilast monotherapy has been considered insufficient in psoriasis vulgaris. However, a clinical trial in which apremilast administration was combined with NB‐UVB phototherapy showed excellent efficacy, with a PASI75 achievement rate of 73% at week 12 and no increase in the incidence of adverse events.^16^ A retrospective study demonstrated increased efficacy of apremilast when combined with phototherapy, other systemic treatments, or biologics, with 81% achieving a PASI75 at 12 weeks.^17^

Phototherapy is an effective and relatively reasonable treatment for psoriasis vulgaris with few adverse events. NB‐UVB is particularly effective for the treatment of psoriasis, with rapid lesion clearance, fewer excessive erythema episodes, and longer remission periods.^5^ NB‐UVB has two modes of action: induction of apoptosis and induction of antigen‐specific immunosuppression, likely regulatory T (Treg) cell induction. The NB‐UVB–induced depletion of pathogenically relevant T cells is the result of the induction of apoptosis.^5^ Bath‐psoralen UV‐A therapy induced circulating Foxp3^+^ Treg cells in 10 patients with psoriasis who were first treated with phototherapy.^18^ In another study, the Treg cell levels were analyzed in 68 patients before and after phototherapy.^19^ Although the Treg cell levels were not increased by phototherapy in any of the 68 patients, they were significantly increased in patients who initially had <4.07% Treg cells, which was defined as the mean in the controls. Assessment of the Treg cell function before and after phototherapy revealed a significantly lower Treg cell function ratio in patients with psoriasis than in age‐matched controls before therapy, which was significantly increased following phototherapy, thereby restoring Treg cell function to almost normal levels. These findings suggest that successful phototherapy not only increases the Treg cell levels but also restores its function.^19^

Phototherapy has many advantages in the treatment of psoriasis, but continued use is inconvenient because the effects of the therapy appear only after a long time, and patients are required to frequently visit the clinic for irradiation. Therefore, the combination of phototherapy with other modalities should be considered. In this study, the combination of apremilast and phototherapy demonstrated sufficient therapeutic effects in psoriasis despite the reduced frequency of visits to the clinic, which may have considerable implications in clinical practice.

We compared the efficacy and safety of apremilast and phototherapy combination therapy with those of phototherapy as monotherapy in patients with moderate to severe psoriasis vulgaris. The primary end point was PASI improvement from baseline to 8 weeks; the combination of apremilast and phototherapy achieved a higher rate of improvement than with monotherapy. Moreover, if the analysis set was the PPS, the PASI improvement rate was significantly higher in the combination therapy group than in the monotherapy group when the missing values were supplemented with or without LOCF.

In the combination therapy group, the BSA percentage at baseline did not affect the PASI improvement rate; however, in the monotherapy group, the PASI improvement rate decreased when the baseline percentage of BSA was higher. This may indicate that monotherapy is not sufficiently effective in patients with a high BSA ratio at the start of the treatment and that the effects of apremilast complement the limitations of the phototherapy monotherapy with NB‐UVB.

The PASI score, change from baseline, and rate of change in the BSA improved over time in both of the treatment groups, suggesting that phototherapy as monotherapy and in combination with apremilast is effective in the treatment of psoriasis vulgaris. The rates of PASI75 achievement and sPGA0/1, which are frequently used as end points in clinical trials, tended to be higher at 4 weeks and significantly higher at 8 weeks in the combination therapy group than in the phototherapy monotherapy group, suggesting that combined treatment with apremilast and phototherapy may be effective against psoriasis over a shorter period.

In the present study, the EQ‐5D‐5L, DLQI (indicators of the patient's quality of life), and VAS (an indicator of itchiness) scores did not differ significantly between the two groups but tended to decrease in the combination therapy group. These findings suggest that an 8‐week treatment regimen may not be sufficient in patients for an improvement in their subjective assessment of psoriasis, and longer treatment periods may be necessary.

Our findings indicate that phototherapy as monotherapy was insufficient in patients with high baseline BSA, and concomitant administration of apremilast improved the outcomes. However, an 8‐week treatment regimen, may be insufficient for inducing subjective improvement.

## CONFLICT OF INTEREST

This research was funded by Amgen K.K. Tokyo, Japan. Akimichi Morita has received research grants, consulting fees, and/or speaker's fee from AbbVie, Amgen, Boehringer Ingelheim, Bristol Myers Squibb, Celgene, Eli Lilly, Eisai, Janssen, Kyowa Hakko Kirin, LEO Pharma, Maruho, Mitsubishi Tanabe, Nichi‐Iko, Nippon Kayaku, Novartis, Pfizer, Sun Pharmaceutical Industries Taiho Pharmaceutical, Torii Pharmaceutical, Ushio and UCB Pharma. Yukie Yamaguchi declares receiving research grants, and/or consulting fees, and/or speaker's fees from AbbVie, Amgen, Astellas, Boehringer Ingelheim, Eisai, Eli Lilly, Janssen, Kyowa Kirin, LEO Pharma, Maruho, Mitsubishi Tanabe, Novartis, Sun Pharmaceutical Industries, Taiho Pharmaceutical, Torii Pharmaceutical, and UCB Japan. Chiharu Tateishi has received research grant, consulting fee, and/or speaker's fee from Amgen. Daisuke Hayashi has received research grant, consulting fee from Amgen. Yuko Watanabe has received speaker's fee from AbbVie, Eli Lilly, Maruho, Novartis, Taiho Pharmaceutical and UCB Pharma. Koji Masuda has received research grants from Eli Lilly Japan. Daisuke Tsuruta has received research grants and/or speaker's fee from Abbvie, Boehringer Ingelheim, Bristol Myers Squibb, Eli Lilly, Eisai, Janssen, JIMRO Co., Ltd., Kyowa Hakko Kirin, Maruho, Mitsubishi Tanabe, Nippon Kayaku, Novartis, Sun Pharmaceutical Industries, Pfizer, Taiho Pharmaceutical, Teijin Limited, Torii Pharmaceutical, Tsumura & Co. and UCB Pharma. Norito Katoh has received honoraria as a speaker/consultant for Sanofi, Maruho, Abbvie, Ely‐Lilly Japan, Leo Pharma, Jansen Pharma, Mitsubishi Tanabe Pharma, Kyowa Kirin, Celgene Japan and has received grants as an investigator from Maruho, Ely‐Lilly Japan, Sun Pharma, Taiho Pharmaceutical, Torii Pharmaceutical, Boehringer Ingelheim Japan Mitsubishi Tanabe Pharma, Kyowa Kirin, and Leo Pharma. Kyoko Ikumi, Aya Yamamoto, Haruna Nishihara, Yukihiko Watanabe and Ayano Maruyama have nothing to disclose.
